# Neuroborreliosis Mimicking Leptomeningeal Carcinomatosis in a Patient With Breast Cancer: A Case Report

**DOI:** 10.1177/2324709614529417

**Published:** 2014-03-28

**Authors:** Stefanie Fischer, Johannes Weber, Isabelle Senn-Schönenberger, Thomas Cerny, Thomas Hundsberger

**Affiliations:** 1 Cantonal Hospital, St Gallen, Switzerland; 2 Private Practice, St Gallen, Switzerland

**Keywords:** leptomeningeal carcinomatosis, neuroborreliosis, breast cancer, leptomeningeal metastases

## Abstract

Leptomeningeal carcinomatosis is a serious complication of advanced cancer. Various clinical manifestations may present, such as headache, nausea, seizures, cranial neuropathies. In this article, we report the case of a 65-year-old woman with metastatic breast cancer who was admitted to hospital suffering from facial palsy, which was suspected to be caused by leptomeningeal tumor infiltration. Magnetic resonance imaging (MRI) scans of the head and spine showed meningeal enhancement of the facial nerve, conus medullaris, and fibers of the cauda equina, which were radiologically interpreted as leptomeningeal carcinomatosis. Assessment of cerebrospinal fluid found no malignant cells but investigation for infectious diseases established the diagnosis of neuroborreliosis. Antibiotic treatment with doxycycline was performed. After completion of treatment, follow-up MRI scans found complete regression of meningeal enhancement. Several months later, the patient is still in good condition and without neurological symptoms. Hence, initial diagnosis of leptomeningeal carcinomatosis was rejected. This case report should alert oncologists to carefully rule out infectious diseases before leptomeningeal carcinomatosis is diagnosed. Cerebrospinal fluid analysis is strongly recommended due to low specificity of MRI images in this regard.

## Introduction

Leptomeningeal metastases are a rare but usually severe complication of advanced cancer. Most common solid tumor causes of leptomeningeal metastases include breast, lung, gastrointestinal malignancies, and melanoma.^1^ Clinical features are diverse and may involve headache, nausea, cognitive decline, multiple cranial neuropathies, cerebellar dysfunction, radiculopathy, seizures, pain, and other symptoms.^2^

Typically, development of symptoms is subacute over a period ranging from days to several weeks. Diagnosis of leptomeningeal metastases is usually made by magnetic resonance imaging (MRI) scan of brain and spine and cerebrospinal fluid (CSF) analysis. MRI findings suggestive of leptomeningeal carcinomatosis in a given patient with or without a known cancer include leptomeningeal, subependymal, dural, or cranial nerve contrast enhancement.^1,3^ CSF usually shows high opening pressure, low glucose concentration, high protein level, lymphocytic pleocytosis, and positive cytology for malignant cells.^2,3^

Treatment strategies depend on symptoms, performance status, and the overall prognosis of the patient. The tumor type and systemic disease burden should also be taken into account for clinical decision making. Focal radiotherapy and intrathecal and systemic chemotherapy are usually applied.^4^ To control increased intracranial pressure, pain, and other symptoms, corticosteroids such as dexamethasone are frequently used.

Differential diagnosis of other neurological or infectious central nervous system (CNS) diseases can sometimes be difficult as symptoms of leptomeningeal metastases are diverse.

We here present the first case report of a patient suffering from metastatic breast cancer and a neuroborreliosis as a differential diagnosis of leptomeningeal metastases.

## Case Report

A 65-year-old woman with a history of a metastasized breast cancer (initial diagnosis in 2003) was admitted to hospital by the end of August 2013 with a newly developed peripheral facial palsy on the right side of the face. Several months before admission, she had been under a metronomic therapy with cyclophosphamide and methotrexat with a stable tumor situation. Metastases of the lung and pleura, a solitaire metastasis in the liver, as well as multiple tumor manifestations in vertebral bodies were known.

**Figure 1.**
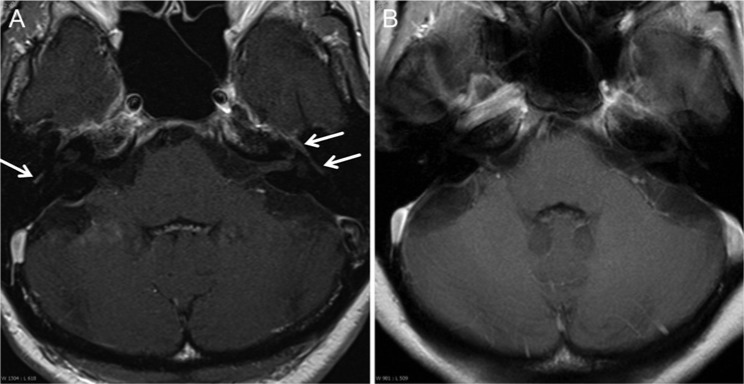
(A) MRI head: axial T1 weighted with contrast agent (August 2013): pathological enhancement of N. facialis on both sides in tympanal segment (arrows), expansion as far as Foramen stylomastoideum (not shown). (B) MRI head after treatment: axial T1 weighted with contrast agent (September 2013): enhancement of N. facialis in the tympanal segment is no longer detectable.

**Figure 2.**
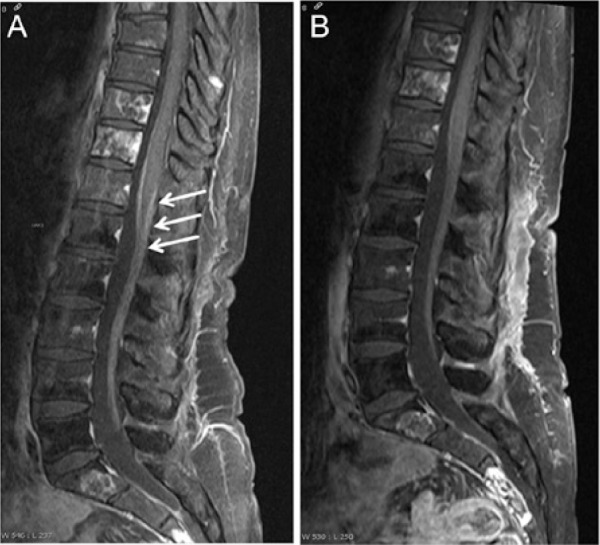
(A) MRI long spine T1 weighted with contrast medium (August 2013): enhancement of Conus medullaris and fibers of the Cauda equina (arrows), disseminated metastases of vertebral bodies. (B) MRI long spine after treatment (September 2013): T1 weighted with contrast agent: the former enhancement of Conus medullaris and Cauda equina can only minimally be seen any longer.

Initial diagnostic suspicion was facial palsy due to a newly developed leptomeningeal carcinomatosis. MRI of the brain and spine were therefore determined and already performed in ambulatory setting. MRI scan of the head showed gadolinium enhancement of the facial nerve on both sides (Figure 1A), and MRI of the vertebral spine revealed a strong contrast enhancement of the conus medullaris and fibers of the cauda equina (Figure 2A).

On admission to hospital, the patient presented a good performance status with normal vital signs and no clinical neurological abnormalities apart from right sided facial palsy.

On enquiring for abnormalities of the skin, the patient reported to have perceived exanthema of the body trunk several weeks ago. She had not mentioned it before as she considered it not to be relevant. At the beginning the lesion was red, apparently increasing in size and showing a garland-like look with a fading in the center soon after. No local pain or itching was reported, and the patient could not remember a tick bite or exposure to other parasites or insects. The exanthema showed a spontaneous complete regression over time. Moreover, the patient complained about a diffuse muscle pain in the last weeks, followed by development of the asymmetry of the face, which she first realized during brushing her teeth.

Laboratory testing showed normal values for hemoglobin and leukocytes, slightly elevated thrombocytes, unimpaired kidney and liver function, and a normal C-reactive protein value.

Due to the history of a possible Erythema migrans, serologic testing for *Borrelia burgdorferi* was performed and found high positivity for specific IgM and IgG antibodies. A lumbar puncture showed moderately elevated total protein concentration, normal glucose level, and pleocytosis of mononuclear cells. Cytologic differentiation found lymphocytes but no carcinoma cells.

A CSF/serum ratio >2 of specific intrathecal antibodies against *B burgdorferi* finally proved the diagnosis of neuroborreliosis.^5,6^

Accordingly, radiotherapy, which was intended due to the suspicion of a leptomeningeal carcinomatosis, was postponed. Antibiotic treatment with doxycycline for 3 weeks was initiated,^7-9^ and the patient was discharged from hospital.

Further clinical assessment showed persisting well-being and a complete regression of the facial palsy without development of new neurologic symptoms during the following weeks.

**Figure 3.**
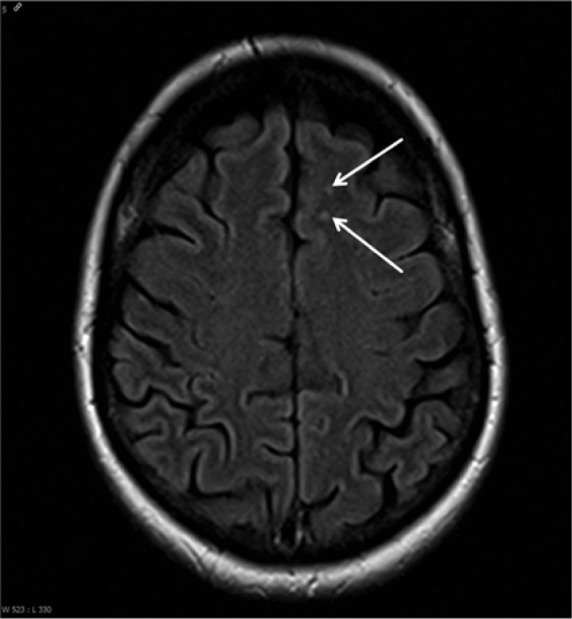
MRI head axial, FLAIR (August 2013): multiple unspecific gliosis spots in the white matter, seen bihemispheric, which can also occur due to neuroborreliosis, exemplarily 2 frontal lesions shown here (arrows).

Follow-up MRI scans of head and spine 6 weeks after initial presentation and after termination of antibiotic treatment demonstrated complete regression of the meningeal enhancement of both facial nerves and of the spinal cord (Figures 1B and 2B). Therefore, we abandoned the diagnosis of a leptomeningeal carcinomatosis and concluded that all former neurological and MR tomographic abnormalities were due to a neuroborreliosis (see also Figure 3).

To date, the patient is still in good condition and stable tumor situation without any new neurological abnormalities.

## Discussion

Borreliosis is the most common tick-borne infection in Europe and the United States. Incidence varies according to geographic region; for Europe, annual incidences of about 70 to 150/100 000 have been reported.^10,11^ Manifestations of Borreliosis can be divided into 3 phases: early localized, early disseminated, and late disease. Characteristic of early localized disease is the typical skin lesion called Erythema migrans. Neurological symptoms of early disseminated Borreliosis may include cranial nerve palsies (most often facial palsy), lymphocytic meningitis, radiculopathy, and peripheral neuropathy. Facial palsy can occur bilaterally and is more often seen in children.^12^ Late disease, which usually occurs months to years after infection, can lead to affection of the joints, encephalopathy, and alterations of the skin such as Acrodermatitis chronica atrophicans.^13^

Leptomeningeal metastases can also cause cranial neuropathies in cancer patients. However, most patients present with pain and nonspecific symptoms such as headache, diffuse weakness, and nausea, which may be related to elevated intracranial pressure.^4,14^ Symptoms may also include seizures, visual disturbances, and incontinence.^14^

Incidence of CNS metastases is generally increasing, mostly because of improved systemic therapies and more long-term survivors of advanced cancer.^15,16^ Metastases of the CNS are common in breast cancer; women with early-stage breast cancer have a long-term risk of about 5% for development of CNS metastases.^17,18^ The majority of CNS metastases in breast cancer patients are parenchymal brain metastases. Additionally, it is estimated that about 11% to 20% of all CNS metastases in breast cancer patients account for leptomeningeal metastases.^19,20^

Because of the diversity of symptoms, neuroimaging with MRI is usually performed for investigation of leptomeningeal carcinomatosis in cancer patients. MRI is highly sensitive and may demonstrate enhancement of the leptomeninges, cranial nerves, ventricular surface, and spinal fibers.^1,3^ However, these changes are nonspecific and careful differential diagnosis is necessary for decision making with regard to treatment as in our patient.

Four similar cases of difficult differential diagnosis between neuroborreliosis und cerebral manifestation of a malignant disease have been reported so far. Schweighofer et al^21^ reported Lyme disease in a patient with chronic lymphocytic leukemia leading to initial suspicion of leptomeningeal infiltration. Antibiotic treatment achieved complete remission of all symptoms. Curless et al^22^ and Walther et al^23^ even presented 2 cases of space occupying cerebral lesions that were initially interpreted as a brainstem tumor of a 15-year-old and as CNS lymphoma, respectively. Both manifestations were also related to neuroborreliosis. Ruitenberg et al^24^ published a case of an older patient with prostate cancer and a painless paraparesis of the legs. Leptomeningeal carcinomatosis was suspected; however, testing for *B burgdorferi* was positive and symptoms completely resolved after antibiotic treatment.

These cases, in addition to the case we present here, show the importance that the diagnosis of leptomeningeal metastases should ideally be based on both MRI scan and CSF analysis. Despite a high sensitivity of MRI, findings of leptomeningeal alteration are mostly nonspecific and can only be suggestive.^4,25^ If risk of lumbar puncture is justifiable, CSF analysis should therefore be performed to confirm suspicion of leptomeningeal metastases and to exclude other differential diagnosis.

We here report the first case of a breast cancer patient with a proven neuroborreliosis with radiological signs being misinterpreted as leptomeningeal carcinomatosis. In conclusion, oncologists should be alerted to think of other differential diagnosis if a patient with an active underlying malignant disease presents with new neurological symptoms. Leptomeningeal metastases as a serious complication mostly arising in later stages of a cancer disease always have to be taken into account. However, infectious diseases such as neuroborreliosis have to be ruled out before clinical decision making.
